# Functional properties of patient-derived melanoma cancer stem cells

**DOI:** 10.1186/2051-1426-3-S2-P309

**Published:** 2015-11-04

**Authors:** Gabriel Nistor, Aleksandra Poole, Hans Keirstead

**Affiliations:** 1 Caladrius Biosciences, Inc., Irvine, CA, USA

## 

Caladrius Biosciences has recently identified biomarkers that may predict clinical efficacy in immunotherapy treatments for melanoma. While numerous clinical trials demonstrated success in long-term survival using immunotherapy agents in various types of malignancies, very few have demonstrated the underlying mechanism of action of these therapies. Caladrius Biosciences is developing an innovative approach to cancer treatment targeting the cells responsible for tumor growth and metastasis, known as cancer stem cells (CSC) or tumor-initiating cells (TIC). The therapy (CLBS20, also known as NBS20) uses purified autologous CSCs, derived from a patient's own tumor and grown as a tumor cell line, as the antigen source of tumor-associated antigens to induce or enhance an anti-tumoral immune response. Using the immune system to target CSCs may provide a complementary or adjunctive therapy that would result in better long-term control, and possibly even a cure, of certain cancers. This approach has shown consistent and compelling results in sequential open-label and randomized Phase II trials for advanced stage melanoma, and is now being tested in a Phase III trial called the Intus Study.

In order to determine whether isolated CSC populations contained antigenic cancer-related mutations, exomic sequencing was performed on tumor cell lines from 5 distinct melanoma tumors using Illumina's sequencing technology. Sequencing results, combined with epigenetic data, indicated the presence of cancer and metastasis-related driver mutations with highly antigenic properties. Identification of these mutated antigens allows us to further study specific T cell activation using mixed lymphocyte reaction assays. Concurrently, we analyzed patient serum samples before and 1 week after 3 weekly injections of CLBS20, using comprehensive high-throughput screening for proteins responsible for immune response, inflammation and angiogenesis. This approach allows us to look into mechanisms of immunosuppression by which tumor cells circumvent native and adaptive immune responses. The analysis of cytokines demonstrated a decrease of serum tumor markers and the reduction of Treg response in the treatment arm, compared to the control arm, and the results suggest a combination of humoral and cell immune responses. A set of biomarkers associated with poor response in the treatment arm was identified. This discovery demonstrates a complex immune response following administration of CLBS20 immunotherapy and is of critical importance for effective prediction of patient response to therapy.

